# From nurse to patient: A breast cancer journey of faith, fear, and endurance

**DOI:** 10.1017/S1478951526102570

**Published:** 2026-04-30

**Authors:** Beulah Joy Damasco

**Affiliations:** Nursing, Bukidnon State University, Philippines

I am a nurse by profession. For years, I have cared for patients and offered reassurance during difficult moments. I believed I understood illness until I became the patient myself. Two years ago, my life changed when I discovered a small lump in the upper portion of my left breast during a routine breast self-examination. As a nurse, I knew the importance of early detection. As a daughter who had lost both parents to cancer, I also knew the fear that comes with uncertainty. That morning, I felt a quiet but overwhelming unease.

The following day, I consulted a surgeon who was also a friend. On that same day, I underwent several diagnostic examinations. Two days later, while in the hospital, I received the results: malignant and invasive breast cancer. In that moment, everything seemed to collapse. I felt numb, then overwhelmed, and finally broken. I silently cried, trying to process what I had just heard. Telling my family especially my children was the hardest part. I worried not only about my illness but about the impact it would have on them. The nights that followed were sleepless and full of worry. My thoughts were filled with fear: Am I going to die soon? What will happen to my family? Will I still be able to care for them?

The following week, I underwent a mastectomy. The days leading up to the surgery were filled with anxiety, but I chose to place my trust in God. I held on to faith as I prepared myself emotionally and physically. I was surrounded by the support of my family, relatives, friends, and colleagues. Their prayers and encouragement became my strength. During the surgery, additional specimens were sent for biopsy to determine the aggressiveness of the cancer. The waiting period that followed felt endless. For 3 weeks, I lived in uncertainty. When the results came, I learned that my cancer was not only invasive but also aggressive. I was triple positive – estrogen receptor positive, progesterone receptor positive, and HER2 positive. I cried again, but this time, I also made a decision. I would fight. I would face the treatments, no matter how difficult they might be.

My treatment journey was long and physically demanding. I underwent 8 cycles of chemotherapy followed by 30 days of radiation therapy. Because my cancer was aggressive, I also received targeted therapy for 18 cycles every 21 days. Chemotherapy brought intense side effects. There were days when my body felt weak and exhausted. I required blood transfusions almost every 2 months. Each cycle tested my endurance. Yet, with every challenge, I reminded myself why I needed to keep going. At present, I continue taking oral chemotherapy, which brings daily side effects and frequent visits to my oncologist. I am not yet cancer-free, and I continue to wait for that moment with hope.

Being both a nurse and a patient transformed how I understand illness and care. Before, I focused on treatment protocols, recovery timelines, and clinical outcomes. Now, I understand the deeper realities of living with cancer – the uncertainty, the emotional burden, the financial strain, and the importance of compassionate support. I have learned that healing is not only about curing disease but also about sustaining hope. The presence of family, the encouragement of friends, the prayers of colleagues, and my faith in God became essential parts of my healing journey.

Today, I take life one day at a time. Cancer has taught me patience, resilience, and gratitude. The journey is not easy – physically, mentally, emotionally, and financially – but I continue to move forward. I trust that God is in control and that He makes no mistakes. My experience has changed me not only as a patient but also as a nurse. I now listen more deeply, empathize more sincerely, and care more holistically. While I continue to wait for the day, I can say I am cancer-free, I choose to live with hope, faith, and courage – one day at a time.

